# Picosecond excitation energy transfer of allophycocyanin studied in solution and in crystals

**DOI:** 10.1007/s11120-017-0417-4

**Published:** 2017-07-28

**Authors:** Reza Ranjbar Choubeh, Ravi R. Sonani, Datta Madamwar, Paul C. Struik, Arjen N. Bader, Bruno Robert, Herbert van Amerongen

**Affiliations:** 1 Laboratory of Biophysics, Wageningen University, Wageningen, The Netherlands; 2 Post-Graduate Department of Biosciences, UGC-Centre of Advanced Study, Sardar Patel University, Bakrol, Anand, Gujarat 388 315 India; 3 Commission of Atomic and Alternative Energy, Institute of Biology and Technology of Saclay, 91191 Gif-sur-Yvette, France; 4 Centre for Crop Systems Analysis, Wageningen University, Wageningen, The Netherlands; 5 MicroSpectroscopy Centre, Wageningen University, Wageningen, The Netherlands; 6 BioSolar Cells, P.O. Box 98, 6700 Wageningen, The Netherlands

**Keywords:** Allophycocyanin crystals, Excitation energy transfer, Phycobilisome, Cyanobacteria, Time-resolved fluorescence spectroscopy

## Abstract

Cyanobacteria perform photosynthesis with the use of large light-harvesting antennae called phycobilisomes (PBSs). These hemispherical PBSs contain hundreds of open-chain tetrapyrrole chromophores bound to different peptides, providing an arrangement in which excitation energy is funnelled towards the PBS core from where it can be transferred to photosystem I and/or photosystem II. In the PBS core, many allophycocyanin (APC) trimers are present, red-light-absorbing phycobiliproteins that covalently bind phycocyanobilin (PCB) chromophores. APC trimers were amongst the first light-harvesting complexes to be crystallized. APC trimers have two spectrally different PCBs per monomer, a high- and a low-energy pigment. The crystal structure of the APC trimer reveals the close distance (~21 Å) between those two chromophores (the distance within one monomer is ~51 Å) and this explains the ultrafast (~1 ps) excitation energy transfer (EET) between them. Both chromophores adopt a somewhat different structure, which is held responsible for their spectral difference. Here we used spectrally resolved picosecond fluorescence to study EET in these APC trimers both in crystallized and in solubilized form. We found that not all closely spaced pigment couples consist of a low- and a high-energy pigment. In ~10% of the cases, a couple consists of two high-energy pigments. EET to a low-energy pigment, which can spectrally be resolved, occurs on a time scale of tens of picoseconds. This transfer turns out to be three times faster in the crystal than in the solution. The spectral characteristics and the time scale of this transfer component are similar to what have been observed in the whole cells of *Synechocystis* sp. PCC 6803, for which it was ascribed to EET from C-phycocyanin to APC. The present results thus demonstrate that part of this transfer should probably also be ascribed to EET within APC trimers.

**Electronic supplementary material:**

The online version of this article (doi:10.1007/s11120-017-0417-4) contains supplementary material, which is available to authorized users.

## Introduction

Cyanobacteria harvest light for photosynthesis via huge membrane-bound, multi-molecular complexes, the phycobilisomes (PBSs). The PBS is a well-organized stack of coloured phycobiliproteins (PBPs) and colour-less linker peptides. The PBPs bind open-chain tetrapyrrole chromophores, which would otherwise be insoluble in the cellular aqueous environment. During the last two decades, it has been shown that PBPs do not just hold chromophores, but also play a vital role in defining the chromophores’ spectral characteristics, which determine the direction, rate and efficiency of excitation energy transfer (EET) [for reviews see (Sidler 1994; Watanabe and Ikeuchi 2013)].

Allophycocyanin (APC) is a red-light-absorbing PBP that covalently binds phycocyanobilin (PCB) chromophores. Together with other proteins such as L_CM_, L_C_ and allophycocyanin-B, APC forms the core of the PBS (Gingrich et al. 1983; Ducret et al. 1998). APC is a trimer (radius: ~11 nm, thickness: ~3 nm) of monomers, each consisting of two peptides, called the α- and β-subunits, both containing a single PCB attached to a conserved cysteine residue.

Monomeric APC has an absorption spectrum similar to that of C-phycocyanin (C-PC). However, when APC monomers form a trimer, the absorption spectrum shows a red-shift of ~30 nm (Lundell and Glazer 1981; MacColl 2004; McGregor et al. 2008) as compared to, for instance, C-PC trimers. This enables APC to be the energy transfer mediator between higher-energy-absorbing peripheral PBPs and lower-energy-absorbing L_CM_ and/or allophycocyanin-B, which further transfer energy to the reaction centre chlorophylls. Upon trimer formation, the PCB chromophore of the α-subunit of one monomer comes close to a PCB, attached to the β-subunit of another monomer. The close proximity of these chromophores is accompanied by a red-shift, although the physical nature of this red-shift is still under debate and various mechanisms have been proposed. Peng et al. (2014) suggested that the unique geometry of PCB in APC causes the red-shift. McGregor et al. (2008) proposed that the red-shift is due to special coupling of the hydrophobic protein microenvironment around αPCB created by the β-subunit. Excitonic interaction between the αPCB and βPCB in adjacent monomers has also been proposed to cause the red-shift (MacColl 2004).

Based on the existing literature, it was concluded in van Amerongen et al. (2000) that EET between both pigments occurs via incoherent downhill Förster transfer from the pigment in the β-subunit to the one in the α-subunit with a time constant in the order of 1 ps. Further equilibration between these pigment pairs within the trimer on the other hand takes tens of ps but it is not expected to be accompanied by significant spectral changes. However, in some recent experiments on APC crystals, we observed some large unexpected spectral changes on a time scale of tens of picoseconds. To study this apparent controversy in more detail, we performed time-resolved fluorescence measurements on APC trimers, purified from *Phormidium* sp. A9DM, in crystalline and solution forms (Sonani et al. 2015).

## Materials and methods

### Protein preparation and crystallization

The APC 660 (referring to the fluorescence maximum at 660 nm) trimers were purified from *Phormidium* sp. A9DM and crystallized as described earlier (Sonani et al. 2015). The crystals did not show any sign of APC 680 fluorescence.

### Steady-state absorption measurements

The steady-state absorption spectrum of APC in 0.05 M phosphate buffer (pH 8.0) was recorded at 25 ± 0.2 °C on a UV–visible spectrophotometer (Specord 210, Analytik Jena AG, Jena, Germany).

### Fluorescence lifetime imaging microscopy (FLIM)

Time-correlated single-photon counting FLIM measurements on crystals were performed similarly as reported in earlier studies in our laboratory on crystals of other photosynthetic complexes (Pascal et al. 2005; van Oort et al. 2008, 2011, 2014). The measurements were done on a Leica SP5X-SMD multi-mode confocal laser scanning microscope using a 63× water immersion 1.2 NA lens. APC crystals were excited using a white-light laser (WLL or supercontinuum laser), which emits a continuous spectrum between 470 and 670 nm, from which individual excitation wavelengths can be selected. Confocal imaging was performed using internal filter-free spectral hybrid detectors. Excitation was performed at 594 nm and fluorescence was detected around 645 nm with a spectral bandwidth of 5–10 nm. Detection was not performed in the fluorescence maximum in order to avoid saturation of the detector. FLIM images with a frame size of 128 × 128 pixels were acquired with an SPC730 TCSPC imaging module (Becker and Hickl, Berlin, Germany). The images were analysed with the SPCImage software (Becker and Hickl, version 3.2.3.0, Berlin, Germany).

Each pixel contains a time trace of the fluorescence emission up to several nanoseconds. The size or scale of the image is 246 × 246 μm. The time-resolved fluorescence of each pixel of the FLIM image was fitted with a single exponential decay:

1 $$a \times \exp \left( {\frac{{ - t}}{\tau }} \right),$$

where $a$ is the amplitude, $\tau$ is the fluorescence lifetime and t is the time after excitation. Each pixel in the FLIM image is colour-coded, using SPCImage, according to the fluorescence lifetime of that pixel. The lifetimes are also presented in a histogram in which the horizontal axis represents the lifetime in picoseconds and the vertical axis the corresponding pixel frequency of these lifetimes. Only those pixels were selected for which the peak was at least 40% as high as the brightest pixel in the image in order to obtain a good signal-to-noise ratio. A typical maximum peak value was ~700 photons. The lifetimes were also calculated after binning each pixel with the 24 surrounding pixels (average of in total 5 × 5 pixels).

### Time-resolved fluorescence spectroscopy

Time-resolved fluorescence measurements were performed with a picosecond streak camera at room temperature (van Stokkum et al. 2008; Tian et al. 2011; Chukhutsina et al. 2015) and a laser repetition rate of 4 MHz. The APC crystals and protein solution were put on thin glass plates and were excited using a Nikon CFI Plan Apo Lambda ×10 objective lens with an excitation wavelength of 590 nm and a power of 3–7 nW. The excitation of APC crystals was achieved in the following way. The sample was scanned with the focused laser beam and the sudden detection of an intense fluorescence signal that would disappear again by a slight movement of the excitation light demonstrated that an APC crystal was being excited.

The images were corrected for the wavelength dependency of the detector and then sliced into 5-nm-wide time traces. The fluorescence kinetics of APC crystals were recorded with a 800 and 2000 ps time window and those of APC proteins in solution with a time window of 2000 ps.

Global and target analyses were performed as described in Tian et al. (2012) and Chukhutsina et al. (2015), using Glotaran (Snellenburg et al. 2012) and the TIMP (Mullen and van Stokkum 2007) package for R.

The average fluorescence lifetime for different detection wavelengths was calculated according to

2 $$\frac{{ \sum \nolimits_{{\text{i}}} \tau _{{\text{i}}} \times {\text{DAS}}_{{\text{i}}} (\lambda )}}{{ \sum \nolimits_{{\text{i}}} {\text{DAS}}_{{\text{i}}} (\lambda )}},$$

where $\lambda$ is the wavelength of detection, ${\text{DAS}}_{{\text{i}}} (\lambda )$ is the ith decay associated spectrum (DAS) obtained from global analysis and $\tau _{{\text{i}}}$ is the corresponding lifetime. The time-zero spectrum was obtained by adding all the DAS that were obtained from the global analysis for one sample. This sum spectrum represents the fluorescence spectrum directly after excitation and relaxation to the Q_y_ states if no additional fast relaxation processes occur that are not captured by the fitting procedure. All the DAS presented in this work are normalized to the maximum of the corresponding time-zero spectrum unless stated otherwise.

## Results and discussion

### Steady-state absorption spectrum and chromophore conformation

**Fig. 1:**
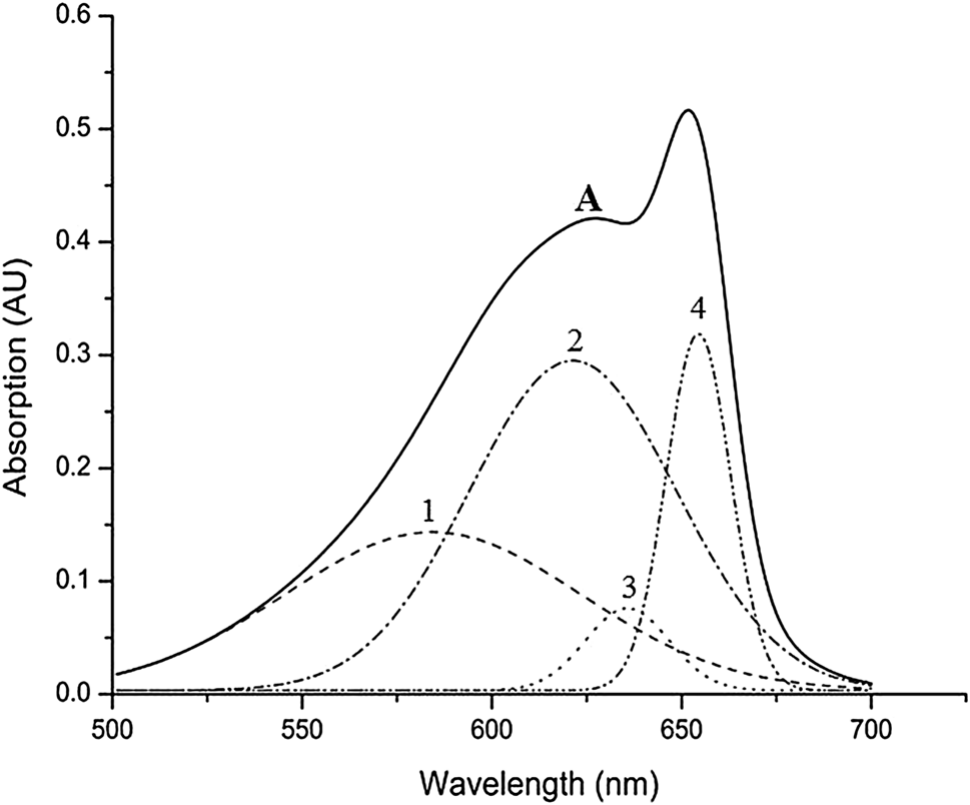
Absorption spectrum (*A, intact line*) of purified Phormidium APC. Gaussian decomposition components of the APC absorption spectrum are represented by the *dashed lines* (1–4)

In Fig. 1, the absorption spectrum of APC is shown together with its Gaussian decomposition, showing four separate components (black dashed lines, 1–4). The absorption spectrum shows a sharp absorption band at 653 nm with a broad shoulder band centred at 620 nm. APC contains two PCBs: one in the α-subunit bound to α81Cys and another in the β-subunit bound to β81Cys (hereafter αPCB and βPCB, respectively) [(Sonani et al. 2015), PDBID: 4RMP]. It is well established that the APC phycocyanobilins with a high deviation from co-planarity of their four pyrrole rings absorb light at shorter wavelengths than PCBs with a low deviation (Peng et al. 2014). PCB contains four pyrrole rings designated as A, B, C and D, and it is connected to the protein via its A ring (Fig. S1a). In *Phormidium* APC, the deviation from co-planarity of the rings in βPCB is higher than that in αPCB (Fig. S1b, c) [(Sonani et al. 2015), PDB ID: 4RMP]. Therefore, it is most likely that the Gaussian absorption components 1 and 2 are due to βPCB, whereas the other two (3 and 4) should be contributed by αPCB, which is in agreement with earlier assignments [see, e.g., (van Amerongen et al. 2000)]. The two chromophores in an αβ-monomer are separated by more than 50 Å; however, the αPCB of one monomer is only 21 Å away from the βPCB of the adjacent monomer within the APC trimer. APC trimers contain three such pairs of spatially clustered low (αPCB) (absorption peak at ~630 and ~653 nm)- and high (βPCB) (absorption peak at ~575 and ~620 nm)-deviation (from co-planarity) chromophores and all αPCBs are arranged more towards the periphery of the trimer ring as compared to βPCB (Fig. S2).

### FLIM

**Fig. 2:**
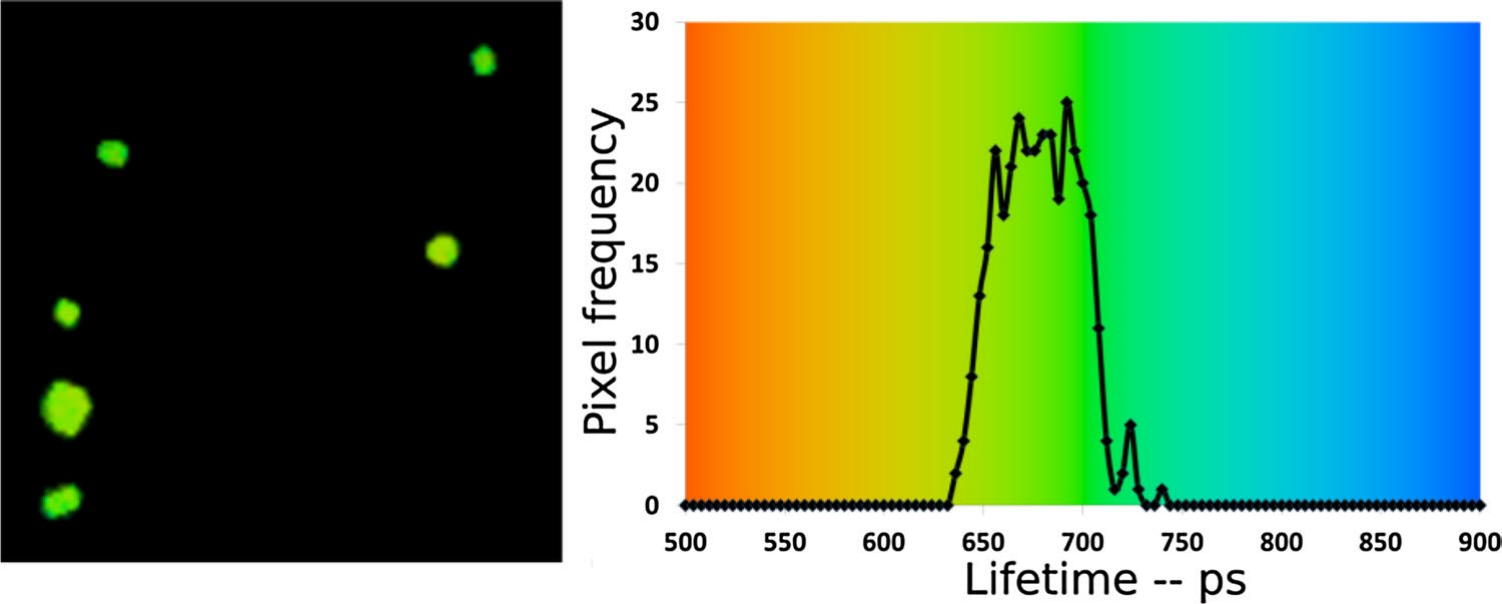
FLIM image of APC crystals (*left*) and the corresponding histogram of the lifetimes (*right*). The FLIM image is made up of 128 × 128 pixels. The image of the crystals roughly contains ~400 pixels. The size of the FLIM image is 246 × 246 μm. The excitation and detection wavelengths are 594 and 645 nm, respectively

We studied the fluorescence kinetics of APC trimers (in crystals) by FLIM and time-resolved and spectrally resolved streak-camera measurements. The results of the FLIM measurements on APC crystals are presented in Fig. 2. The crystals were excited at 594 nm and the fluorescence was recorded at 645 nm. One lifetime was enough to fit the kinetics per pixel. The fitted fluorescence lifetime for the crystals mainly ranged from 650 to 700 ps and the crystals showed a homogeneous lifetime distribution (see the histogram in Fig. 2). The fluorescence lifetime for different detection wavelengths was also calculated using the global analysis of streak-camera images and the results are presented in Fig. S3.

### Time-resolved fluorescence measurements on APC crystals and APC protein solution

**Fig. 3:**
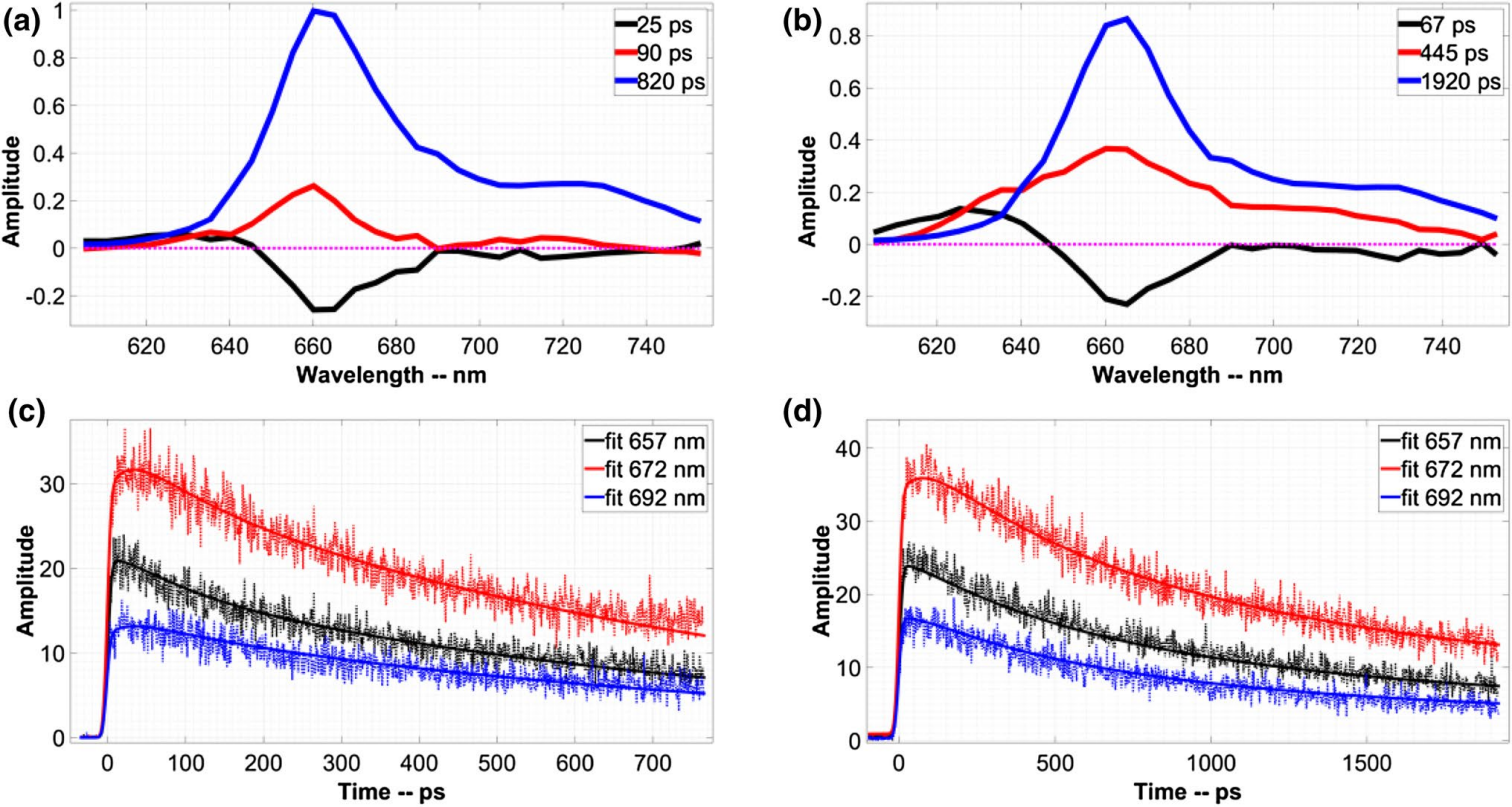
**a, b** DAS obtained from the global analysis of fluorescence data of APC crystals and APC protein solution as measured with the streak camera are shown in **a, b**, respectively. The excitation wavelength was 590 nm. The DAS were normalized to the maximum of the time-zero spectrum. **c, d** Selected measured and fitted time traces of APC crystals and APC protein solution. The *numbers* in the *legends* indicate the detection wavelength. The *solid lines* represent the fits to the time traces

APC crystals and the APC protein solution were subsequently measured with the streak-camera setup and the data were globally analysed (see “Materials and methods”). In all cases, three lifetimes were required to fit the data, in contrast to the FLIM data, where the signal-to-noise ratio was much lower and the time resolution was less. The fitting results of APC crystals and of APC protein solution are presented in Fig. 3a, b. Figure 3c, d shows the selected measured and fitted time traces of APC crystals and APC protein solution, respectively. Each DAS is scaled according to the maximum of the estimated time-zero spectrum. The 25 ps DAS for the crystals and the 67 ps DAS for the protein solution have very similar characteristics, showing a broad positive band below 650 nm and a negative band peaking between 660 and 665 nm. Based on the spectral differences between βPCB and αPCB described above, we assign the 25 and 67 ps DAS in Fig. 3 to EET from βPCB to αPCB. Also in Holzwarth et al. (1990) using time-resolved fluorescence spectroscopy, the authors obtained a 25 ps DAS with a positive peak at 650 nm and a broad negative peak at 660–690 nm. The broadness of the negative peak was explained by a broad range of conformations due to a lack of linker peptides. Time-resolved measurements on wild-type (WT) *Synechocystis* sp. PCC 6803 revealed a 43 ps DAS with a similar shape (see SI and Fig. S4a).

In Fig. 3, the 90 ps DAS of the crystals and the 445 ps DAS of the APC protein solution showed positive maxima at 660 and 660–665 nm, respectively. These positive DAS represent only decay of fluorescence. In Holzwarth et al. (1990), a 720 ps DAS was observed with a broad positive peak centred at 660 nm together with a third DAS with a lifetime of 1700 ps, also centred at 660 nm. The presence of such a broad band together with the presence of different long lifetimes was ascribed to conformational heterogeneity. However, the 90 ps DAS we obtained has a narrow shape that suggests a well-defined conformation of the corresponding chromophores.

In Fig. 3, the 820 and 1920 ps DAS of crystals and protein solution, respectively, showed a positive peak at 660–665 nm, which reflects fluorescence decay. Both DAS reflect a well-defined conformation and have the same shape (Fig. S4c).

In the literature, it is generally reported that EET between spectrally different chromophores in APC protein solution occurs with a time constant of ~1 ps or less (van Amerongen et al. 2000). Although slow processes were reported before, they were not assigned to EET; for example, as discussed earlier, the 25 ps DAS obtained in Holzwarth et al. (1990) was assigned to the decay from the upper excitonic level to the lower excitonic level. In Zhang et al. (1998), both time-resolved isotropic and anisotropic fluorescence spectroscopy revealed, amongst other components, a 40 ps component. The authors assigned the 40 ps lifetime to EET between β84 PCB chromophores in the centre of the APC trimer after exciton localization had occurred within several picoseconds. However, it is not clear how the ~40 ps lifetime could be observed in the isotropic measurements. In Beck and Sauer (1992), one-colour pump–probe experiments resolved, amongst other components, a 45 ± 10 ps decay component between ~590 and ~650 nm, with positive amplitude and no negative amplitude at longer wavelengths. The high power of 5–8 mW that was used, potentially leading to singlet–singlet annihilation, could have been the reason for the absence of a rise term (a negative amplitude) at longer wavelengths.

### Target analysis

**Fig. 4:**
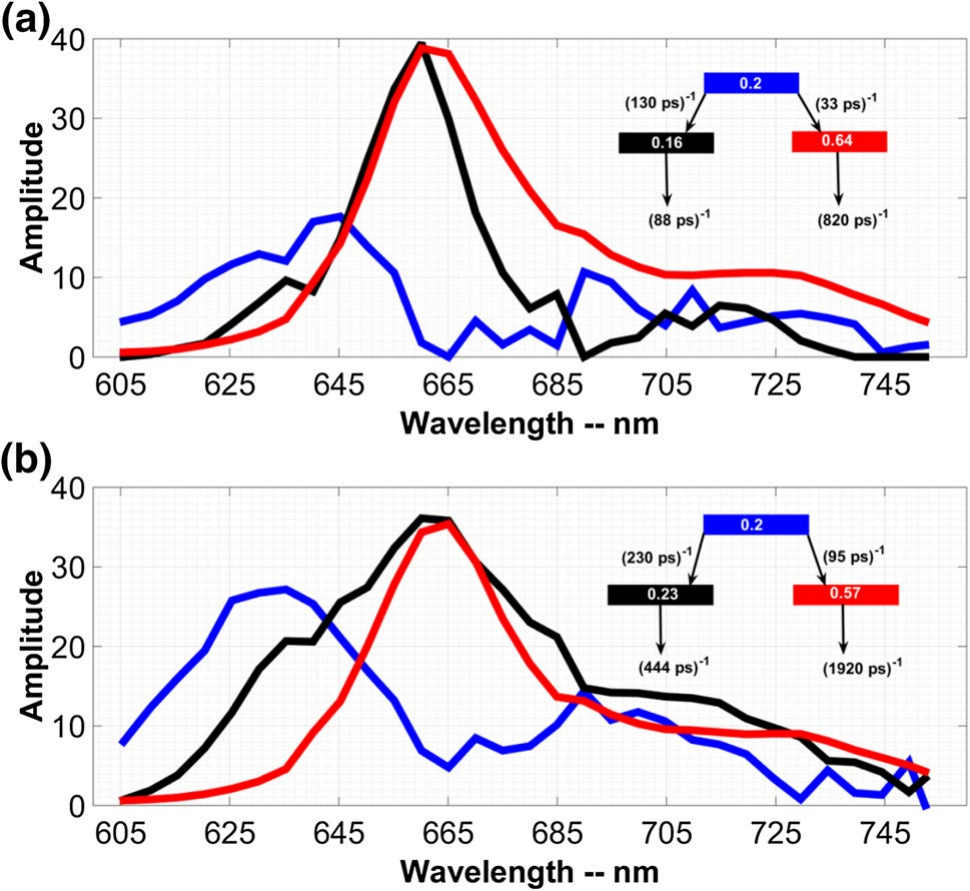
Three species-associated spectra (SAS) as obtained from target analysis are shown for APC crystals and protein solutions in** a**,** b**, respectively. The model used is shown in the figure * inset*. Each compartment represents an emitting species and the *number* written on it is the initial fractional population of excitation. The total initial population is summed to 1. The *colour* of each SAS corresponds to the *colour* of each compartment

To estimate the various spectral components and their contribution to the overall kinetics, we have performed target analysis. Because we resolved three DAS, we also used three compartments. At least two of these should have a different spectrum because the fastest DAS reflects EET between components with different spectra. The target models for crystals and APC protein solution are shown in Fig. 4a, b. The initial fractional populations, which provided the best fits, are given for each compartment.

As we stated before, both ultrafast and slow EET processes occur in APC trimers. This is justified by the presence of ultrafast EET as reported in the literature and the tens of ps EET process in the present work but also reported for instance in Holzwarth et al. (1990). The monomeric unit of APC trimers contains αPCB and βPCB. Regardless which of the αPCB and βPCB has the lower energy, we call them red and blue spectral forms so that the red form represents the lower energy species. According to the absorption decomposition of Fig. 1 initially only the blue forms are excited. If there was only slow EET, then the initial population of the blue form would have been 1, and if there was only ultrafast EET the initial population of the blue form would have been 0 and we would not have been able to observe EET in our measurements as the time resolution of our setup was ~10 ps. The initial population that gives the best fit shows that at time zero the majority of the population resides already in the red spectral form and to a lesser extent in the blue spectral from. This confirms that ultrafast and slow EET co-exist.

Considering the crystal structure, the coexistence of “fast” and “slow” downhill EET is unexpected. We therefore propose that not all the closely spaced α-and βPCBs, dashed orange ovals in Fig. S2, contain a red and blue spectral form, but some of them contain a blue–blue pair. Only after the initial ultrafast EET between red and blue forms has taken place, there is excitation energy transfer from a blue–blue pair to the red form in a neighbouring red–blue pair. Using the obtained initial populations, the percentage of these blue–blue pairs can be estimated. If the ultrafast EET happens only between the blue and red forms, then after ~1 ps the only population remaining in the blue spectral form consists of blue–blue pairs. The time resolution of our measurements as stated before is ~10 ps so we cannot resolve the initial ultrafast EET and the slow EET occurs between the blue–blue and red–blue forms after the ultrafast EET has already occurred. If there are *n* blue–blue pairs and *m* red–blue pairs, then the ratio of the number of blue spectral forms in the blue–blue pairs to the total number of blue spectral forms is $\frac{{2n}}{{2n+m}}.$ This ratio should be the same as the initial population of the blue–blue pairs, which is 0.2 in Fig. 4. This results in $m=8n,$ meaning that ~10% of the closely spaced α- and βPCBs are blue–blue pairs and ~90% are red–blue pairs.

In Fig. 4a, b, the ratio of the rate of EET from the blue to the red compartment to the rate of EET from the blue to the black compartment was made equal to the ratio of the initial populations of the red and black compartments. For example, in Fig. 4a the initial population of the red compartment is 4 times larger than that of the black compartment, so the rate of EET from the blue compartment to the red compartment is 4 times faster than the rate of EET from the blue compartment to the black compartment.

In Fig. 4a, the blue compartment transfers its excitation energy to the black and red compartments with the rates of (130 ps)^−1^ and (33 ps)^−1^, respectively; as a result, the blue compartment decays with a rate of (26 ps)^−1^. The black and red compartments in turn decay to the ground state with the rates of (88 ps)^−1^ and (820 ps)^−1^, respectively.

In Fig. 4b, the blue compartment transfers its excitation energy to the black and red compartments with the rates of (230 ps)^−1^ and (95 ps)^−1^, respectively. The blue compartment then decays with an overall rate of (67 ps)^−1^. The black and red compartments in turn decay to the ground state with the rates of (444 ps)^−1^ and (1920 ps)^−1^, respectively.

The area under the SAS is proportional to the radiative rate of the corresponding species multiplied by an instrument-dependent factor (Loefroth 1986; Holzwarth et al. 1987). In Fig. 4a, the area under the red SAS is ~1.6 times larger than that of the black SAS. One would then expect that the red SAS decays with a faster radiative rate than the black SAS. However, it is the black SAS that decays ~9 times faster. This indicates that the origin of the short lifetime of the black SAS must be non-radiative in nature. The same argument applies to the SAS in Fig. 4b, and the black SAS has a ~1.33 larger area than the red SAS; however, it decays ~4.3 times faster. This indicates that the origin of the shorter lifetime of the black SAS is also non-radiative.

At the moment, we can only speculate why not all blue forms are in close contact with a red form. One possible reason is that not all pigment-binding sites are occupied by pigments, which prevents fast EET between closely coupled pigments and only transfer to more distant pigments can occur on a time scale of tens of picoseconds. Although we cannot completely rule out this possibility, it seems more likely that the same bilin molecules can adopt both a blue and a red conformation in the APC trimers. As was already discussed above, a switch from blue to red occurs upon the oligomerization of monomers into trimers and may be linked to a change in planarity of the bilin molecules. Possibly, an equilibrium exists between such blue and red forms as was also observed for chlorophyll *a* molecules in plant light-harvesting complexes (Passarini et al. 2010; Krüger et al. 2011). It was demonstrated with the use of single-molecule fluorescence experiments that the fluorescence maximum of LHCII, the major light-harvesting complex of plants, can switch from a form that has a fluorescence maximum around 675 nm to a form with a maximum far above 700 nm on a time scale of seconds to tens of seconds (Krüger et al. 2011). Alternatively, the presence of a dynamic equilibrium between the monomeric and trimeric forms may lead to the observed heterogeneity although this seems less likely in the case of crystallized APC. Finally, we cannot rule out that heterogeneity in the excitonic coupling in a minor fraction of the APC trimers may lead to some heterogeneity in the equilibration kinetics.

In conclusion, we have measured excitation energy transfer in APC when present in trimeric form in solution and when crystallized. Most of the EET between blue and red spectral forms occurs within several picoseconds as expected from the crystal structure, but there is also ~10% of the excitations that are transferred on a time scale of tens of picoseconds, meaning that not all supposedly red pigments have adopted a “red conformation”. The rate of EET between the blue and red forms differs for solution and crystal and apparently the crystal form is not exactly the same as the solution form. This might also explain why the fluorescence lifetimes are not identical. The “slow” blue-to-red EET component is also observed in the whole cells of WT *Synechocystis* sp. PCC 6803. This component was previously ascribed to EET from C-PC to APC but according to the present results it might also be partly due to EET within APC.

## Electronic supplementary material

Below is the link to the electronic supplementary material.

Supplementary material 1 (DOCX 617 KB)
