# Supplementary material for: Picosecond excitation energy transfer of allophycocyanin studied in solution and in crystals
Source: Photosynth Res. 2017 Jul 28;135(1):79–86. doi: 10.1007/s11120-017-0417-4 (PMC5783994; doi:10.1007/s11120-017-0417-4)
Supplement: Supplementary file 1 — Supplementary material 1 (DOCX 617 KB) [file 11120_2017_417_MOESM1_ESM.docx]

# Supplementary Information

Picosecond excitation energy transfer of allophycocyanin studied in solution and in crystals

Reza Ranjbar Choubeh^1, 6, #^, Ravi R. Sonani^2, 3, #^, Datta Madamwar^2,^ *, Paul C. Struik^4^, Arjen N. Bader^1, 5^, Bruno Robert^3,^ *, Herbert van Amerongen^1, 5,^ *

^1^Laboratory of Biophysics, Wageningen University, Wageningen, The Netherlands

^2^Post-Graduate Department of Biosciences, UGC-Centre of Advanced Study, Sardar Patel University, Bakrol 388 315, Anand, Gujarat, India

^3^Commission of Atomic and Alternative Energy, Institute of Biology and Technology of Saclay, 91191 Gif-sur-Yvette, France

^4^Centre for Crop Systems Analysis, Wageningen University, Wageningen, The Netherlands

^5^MicroSpectroscopy Centre, Wageningen University, Wageningen, The Netherlands

^6^BioSolar Cells, P.O. Box 98, 6700 AB Wageningen, The Netherlands

^#^Equal Contribution

*Corresponding Authors

#

## Methods

Wild-type *Synechocystis* sp. PCC 6803 cells were measured in a quartz cuvette with an optical length of 1 cm. The OD of the cells was ~0.1 at 670 nm in a cuvette of 1 cm optical length. The cells were excited using 577-nm laser pulses, FWHM 10 nm, and a lens with focal length of 7 cm. The repetition rate of the laser was 4 MHz and the power of the excitation light was ~7 μW. The measurements were performed at room temperature.

The images, recorded by the streak camera with a 800 ps time window, were corrected for the wavelength dependency of the detector and then sliced into 2.5-5 nm wide time traces. Global analysis was performed as described in the main text.

## The chemical structure of phycocyanobilin and spatial structure of allophycocyanin trimers


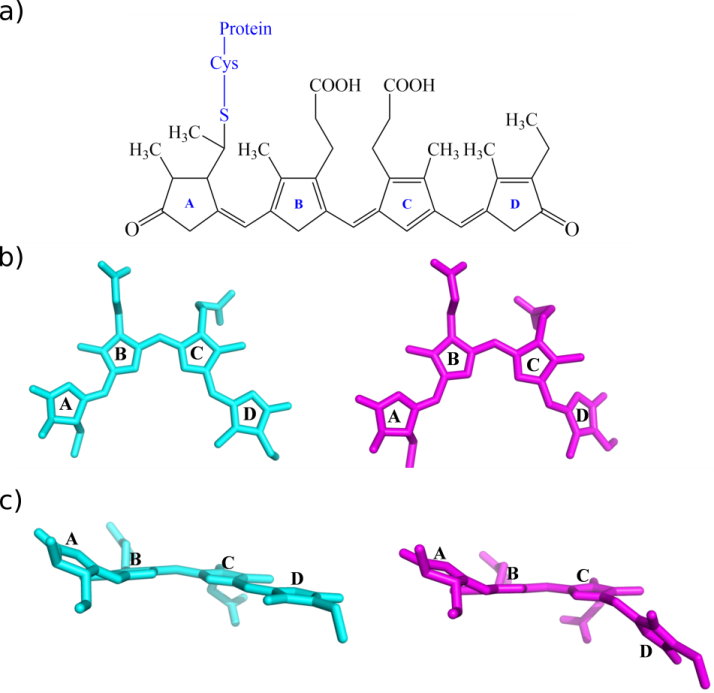


Fig. S1 a) Schematic representation of the PCB chemical structure. b) top view and c) side view of 3D conformation of αPCB (cyan) and βPCB (violate) in the *Phormidium* APC structure (protein data bank code 4RMP). The deviation from co-planarity among the pyrrole rings is notably higher in βPCB than in αPCB


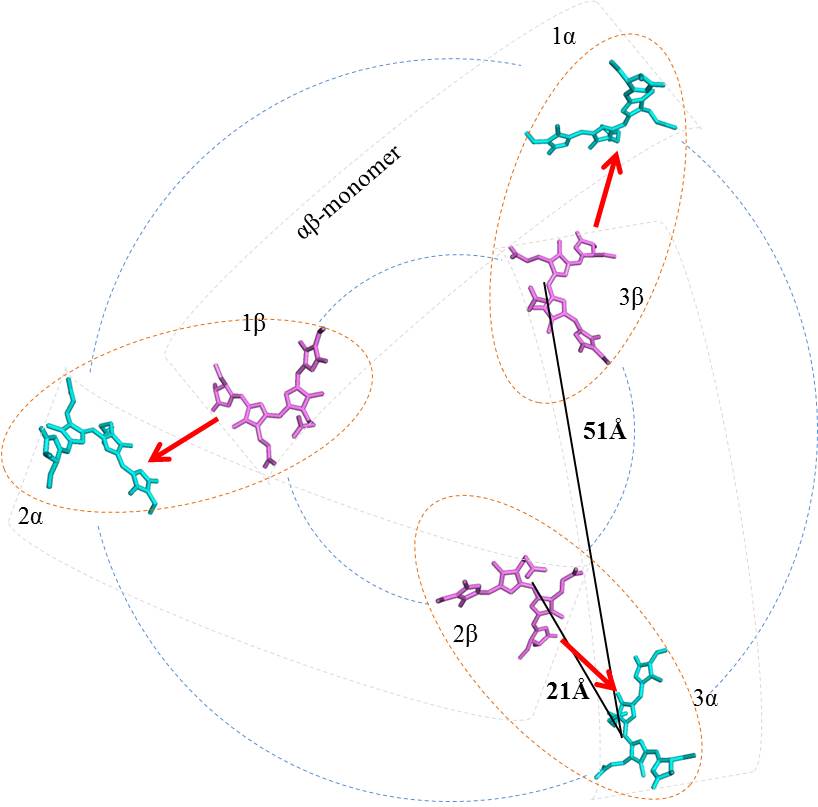


Fig. S2 Spatial arrangement of PCBs in an APC trimer. The αPCBs and βPCBs are shown in cyan and violet, respectively. The dashed-grey shape is a schematic representation of the αβ-monomer. The dashed-orange ovals represent the closely spaced (~21 Å centre-to-centre distance) pigments

## Average lifetimes calculated by global analysis of streak camera data

The fluorescence lifetimes for the APC crystals and protein solution detected with streak camera at 645 nm were calculated as 645 ps and 1.2 ns, respectively (Fig. S3). The fluorescence lifetime of APC crystals detected with the streak camera and with the FLIM setup (detection at 645 nm, see the histogram in Fig. 2) were very similar, which confirms that the time-resolved measurements carried out with the streak camera were indeed performed on APC crystals.


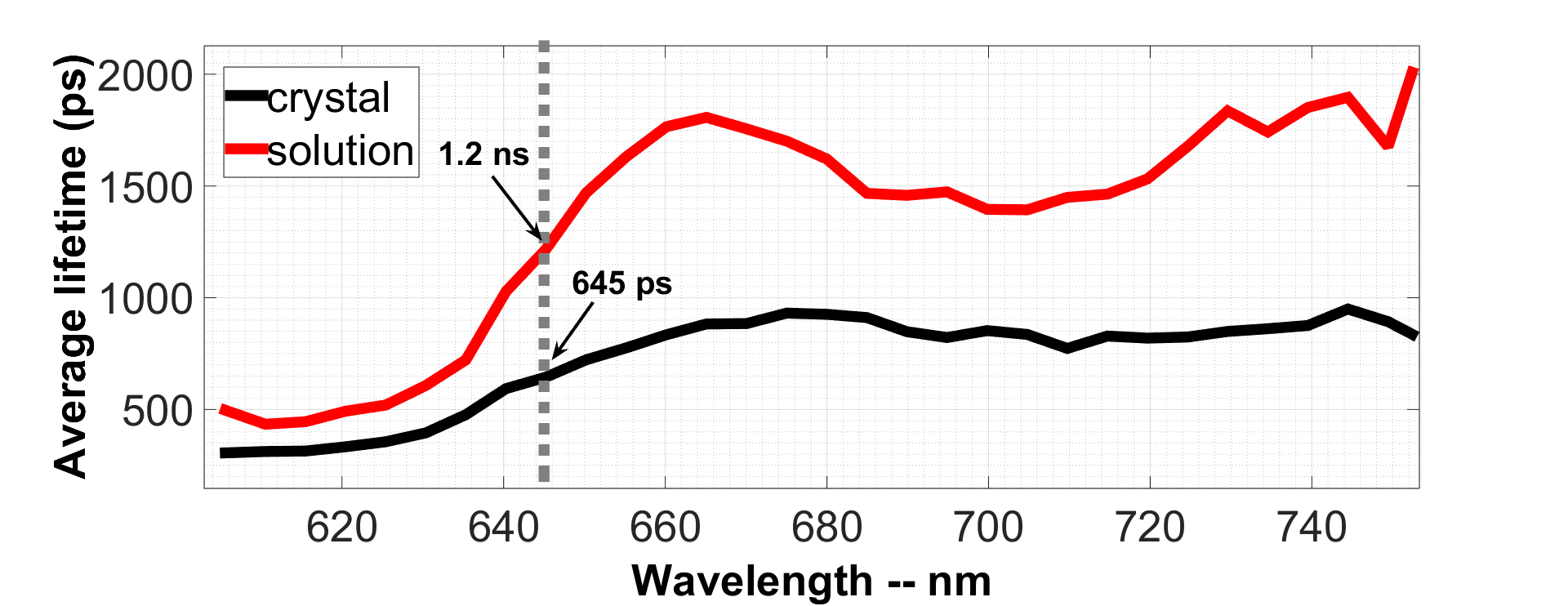


Fig. S3 The fluorescence lifetime of APC crystals and protein solution for different detection wavelengths. The lifetimes were calculated from the global analysis of streak-camera images using equation 2 in the main text. The vertical line shows the 645 nm wavelength that is also the detection wavelength in the FLIM image of Fig. 2

## Comparing the DAS of WT Synechocystis and those of APC in crystal and solution

Time-resolved fluorescence measurements in (Tian et al. 2011) on WT *Synechocystis* sp. PCC 6803 cells and in (Tian et al. 2012) on isolated PBS of WT *Synechocystis* sp. PCC 6803 and isolated PBS from a mutant lacking intermediary and core-distal C-PC hexamers (Ughy and Ajlani 2004) yielded DAS, similar to the 25 ps and 67 ps DAS in Fig. 3. The 39 ps DAS of whole cells (Tian et al. 2011), the 42 ps DAS of isolated WT PBS (Tian et al. 2012), and the 19 ps DAS of isolated PBS lacking the intermediary and core-distal C-PC hexamers (Tian et al. 2012) were assigned to EET from C-PC to APC 660 trimers. The similarity between the DAS of the APC protein solution and the 39 ps DAS of whole cells suggests that the latter can equally well be assigned to EET within APC 660 trimers, at least partly (see below). We also performed time-resolved measurements on WT *Synechocystis* sp. PCC 6803 and obtained a 43 ps DAS from the global analysis that again looks very similar to the 25 ps and 67 ps DAS of crystals and protein solution, respectively (see Fig. S4a). This further supports our suggestion that the 39 ps DAS in (Tian et al. 2011) is at least partly due to EET within APC trimers in the intact cells at room temperature. The difference in the EET lifetimes might be due to a difference in the interaction strength between αPCB and βPCB in the different samples due to the different environments that all these proteins experience, i.e. they are either present in a crystal, solution or a phycobilisome, which might influence the degree of compaction or the number of neighbouring pigments to which EET can take place.

To compare the shapes of the DAS obtained for APC crystals and APC in solution (see Fig. 3), we grouped different DAS and presented them in Fig. S4. In Fig. S4a the 25 ps and 67 ps DAS are normalized to the maximum of their corresponding time zero spectrum. The 43 ps DAS is multiplied by 0.11 to make its amplitude comparable to those of the other two. The rest of the DAS in Figs. S4b-c are normalized to their maximum. In Fig. S4d the time-zero spectrum is shown.


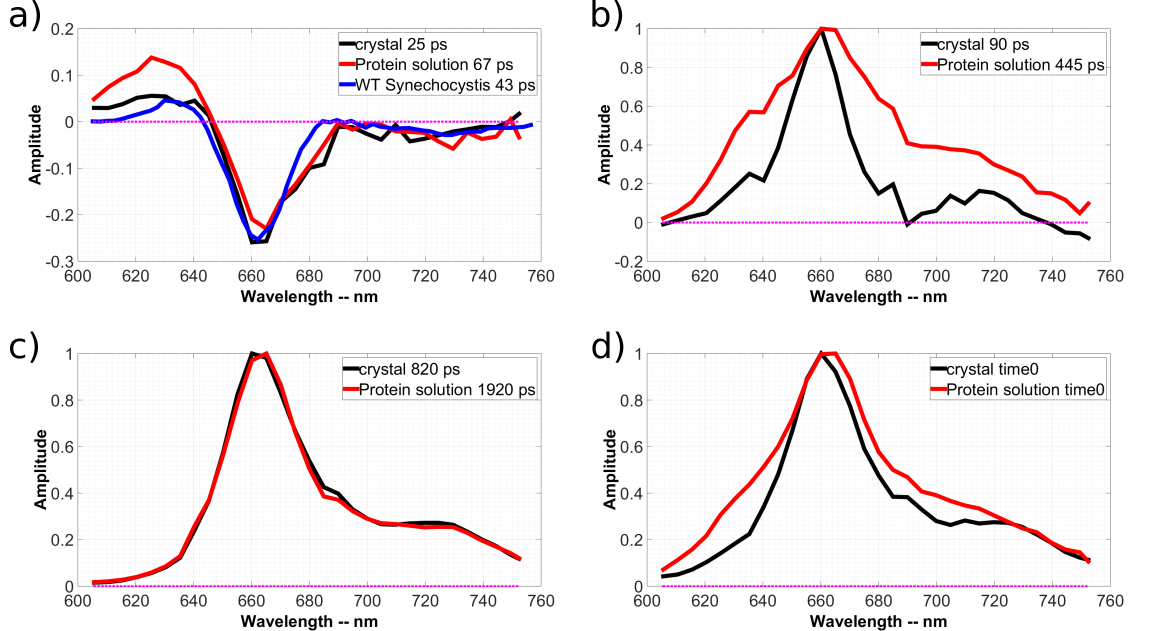


Fig. S4 a-c) Comparison between the DAS of APC crystals and protein solution. In a one of the DAS of WT *Synechocystis* sp. PCC 6803 as obtained with the streak camera is also presented. d) Time-zero spectrum of APC crystals and APC in solution

Tian L, Gwizdala M, van Stokkum IHM, et al (2012) Picosecond Kinetics of Light Harvesting and Photoprotective Quenching in Wild-Type and Mutant Phycobilisomes Isolated from the Cyanobacterium Synechocystis PCC 6803. Biophys J 102:1692–1700. doi: http://dx.doi.org/10.1016/j.bpj.2012.03.008

Tian L, Van Stokkum IHM, Koehorst RBM, et al (2011) Site, rate, and mechanism of photoprotective quenching in cyanobacteria. J Am Chem Soc 133:18304–18311. doi: 10.1021/ja206414m

Ughy B, Ajlani G (2004) Phycobilisome rod mutants in Synechocystis sp. strain PCC6803. Microbiology 150:4147–4156.
